# Influence of Water Regeneration on Chemical and Process Indices in an Energy-Integrated PVC Production Process

**DOI:** 10.3390/polym17121639

**Published:** 2025-06-13

**Authors:** Arelmys Bustamante-Miranda, Eduardo Aguilar-Vásquez, Miguel Ramos-Olmos, Segundo Rojas-Flores, Ángel Darío González-Delgado

**Affiliations:** 1 Chemical Engineering Department, Nanomaterials and Computer Aided Process Engineering Research Group (NIPAC), Universidad de Cartagena, Cartagena 130015, Colombia; abustamantem@unicartagena.edu.co (A.B.-M.); eaguilarv@unicartagena.edu.co (E.A.-V.); 2 Business Administration Department, Grupo de Investigación en Ciencias Administrativas y Seguridad y Salud en el Trabajo (CIASST), Universidad Minuto de Dios-UniMinuto, Cartagena 130001, Colombia; miguel.ramos.o@uniminuto.edu; 3 Institutos y Centros de Investigación, Universidad Cesar Vallejo, Trujillo 13001, Peru; srojasf@ucv.edu.pe

**Keywords:** polymer, inherent safety analysis, wastewater treatment, zero liquid discharge, sustainability, computer-aided process engineering

## Abstract

Water regeneration in PVC production is a key issue to consider, given the high freshwater consumption rate of the process. This research evaluates the inherent safety of poly(vinyl chloride) (PVC) production via suspension polymerization by implementing mass and energy integration strategies in combination with wastewater regeneration under a zero-liquid-discharge (ZLD) approach. The impact of these integrations on process safety was examined by considering the risks associated with the handling of hazardous materials and critical operations, as well as the reduction in waste generation. To this end, the Inherent Safety Index (ISI) methodology was employed, which quantifies hazards based on factors such as toxicity and flammability, enabling the identification of risks arising from system condition changes due to the implementation of sustainable water treatment technologies. Although the ISI methodology has been applied to various chemical processes, there are few documented cases of its specific application in PVC plants that adopt circular production strategies and water resource sustainability. Therefore, in this study, ISI was used to thoroughly evaluate each stage of the process, providing a comprehensive picture of the safety risks associated with the use of sustainable technologies. The assessment was carried out using simulation software, computer-aided process engineering (CAPE) methodologies, and information obtained from safety repositories and expert publications. Specifically, the Chemical Safety Index score was 22 points, with the highest risk associated with flammability, which scored 4 points, followed by toxicity (5 points), explosiveness (2 points), and chemical interactions, with 4 points attributed to vinyl chloride monomer (VCM). In the toxicity sub-index, both VCM and PVC received 5 points, while substances such as sodium hydroxide (NaOH) and sodium chloride (NaCl) scored 4 points. In the heat of reaction sub-index, the main reaction scored 3 points due to its high heat of reaction (−1600 kJ/kg), while the secondary reactions from PVA biodegradation scored 0 points for the anoxic reaction (−156.5 kJ/kg) and 3 points for the aerobic reaction (−2304 kJ/kg), significantly increasing the total index. The Process Safety Index scored 15 points, with the highest risk found in the inventory of hazardous substances within the inside battery limits (ISBL) of the plant, where a flow rate of 3241.75 t/h was reported (5 points). The safe equipment sub-index received 4 points due to the presence of boilers, burners, compressors, and reactors. The process structure scored 3 points, temperature 2, and pressure 1, reflecting the criticality of certain operating conditions. Despite sustainability improvements, the process still presented significant chemical and operational risks. However, the implementation of control strategies and safety measures could optimize the process, balancing sustainability and safety without compromising system viability.

## 1. Introduction

Poly(vinyl chloride) (PVC) is one of the most widely used plastics across numerous industries worldwide due to its multiple properties, low cost, and high versatility, making it a highly economical material for countless applications. Its popularity stems from its physical and chemical characteristics, which make it lightweight, inert, fire-resistant, and highly transparent and durable [1]. PVC is extensively applied in sectors such as construction, automotives, healthcare, textiles, and even in the manufacturing of medical products [2].

However, the most commonly used production method, suspension polymerization, has been criticized for its association with chlorine chemistry and its high water and energy consumption [3]. From an energy standpoint, its production consumes approximately 47 billion kWh per year, equivalent to the output of eight medium-sized nuclear power plants. Moreover, the process demands a significant amount of water during the stages in which water comes into direct contact with raw materials and the final product, such as in PVC synthesis and reactor cooling. It also releases persistent toxic byproducts that tend to bioaccumulate in nearby water sources, thereby affecting both ecosystems and water supplies [4].

In light of these concerns, efforts have been made to develop adsorbent materials that can be directly integrated into wastewater treatment plants, whether in fixed columns, beds, or modular filtration systems, to efficiently and rapidly mitigate industrial environmental loads.

A promising contribution is the work by Mittal et al. [5], involving multi-walled carbon nanotube nanocomposites with thorium oxide ($\mathrm{MWCNTs}/{\mathrm{ThO}}_{2}$). Due to the large surface area of the nanotubes and the strong coordination of metal ions by the oxygen groups in $ThO_{2}$, these materials achieved a Pb(II) removal capacity of 93.5% in just 50 min under the conditions of pH 5.5 and a temperature of 45 °C. However, despite their high efficiency, these materials require further evaluation due to the potential release of thorium and the relatively strict operating conditions of temperature and pH.

In parallel, Khan et al. studied magnetized hydrocarbons derived from sesame waste. These materials demonstrated a Pb(II) adsorption capacity of up to 70% and showed strong affinity for heavy metals due to their oxygen-rich surface, highlighting the rapid performance of biochar-based adsorbents [6]. They stand out for their low cost and the sustainability of their raw materials, which may facilitate their use in treatment systems operating under neutral temperature and pH conditions.

More recently, Wbaidur et al. [7] developed a novel nanocomposite made of silica-coated copper ferrite decorated with oxidized multi-walled carbon nanotubes (CuFe_2_O_4_/oMWCNTs). With a surface area of $182\;m^{2}/g$, this material not only facilitates the adsorption of heavy metals but also enables the removal of cationic dyes such as celestine blue and methylene blue, while demonstrating good regeneration after multiple cycles using organic solvents. This versatility makes it an ideal candidate for plants that treat both metals and dyes simultaneously.

All these strategies demonstrate the potential of advanced adsorbents to enhance the efficiency of water regeneration processes. They not only reduce Pb(II) and dye loads but also directly target the bioaccumulation of persistent compounds, thereby enabling more sustainable and circular treatment systems.

Beyond technological solutions, the regulatory framework reinforces these concepts, as the importance of sustainable development and consumption is now an undisputed priority in the industry. Nevertheless, one of the main obstacles to incorporating sustainability into process and product design is the inherent complexity of the chemical industry [8]. In countries such as Colombia, environmental regulations have evolved to promote safer and more sustainable industrial practices. For instance, Resolution 1256 of 2021, issued by the Ministry of Environment and Sustainable Development, establishes provisions for the reuse of wastewater in both agricultural and industrial sectors. This document also encourages recirculation in industrial processes to minimize the consumption of potable water and the generation of waste [9]. Similarly, Decree 1076 of 2015 in Colombia promotes the reuse of wastewater through plans for the conversion to clean technologies, motivating industries to adopt treatment and reuse systems that contribute to environmental sustainability [10].

In light of these challenges, a set of structured methods based on mass and energy integration has been implemented to provide a comprehensive view of the process. Energy integration is a systematic methodology that enables a better understanding of energy use within a process and, through it, the identification of energy targets and the optimization of heat recovery and energy utilization [11].

Mass integration, on the other hand, is broader and more complex. It allows for an understanding of the overall mass flow within a process, facilitating the establishment of pollution prevention targets [11]. The comprehensive approach it offers makes it essential to develop solutions that, together with strategies such as wastewater regeneration, allow for the partial removal of particles and organic compounds present in industrial effluents, thus reducing the pollutant load. In this way, the recirculation of treated water within the production system is facilitated, reducing not only the demand for potable water and the dependence on external sources, but also the environmental impact caused by the dispersion of toxic substances in effluents from this industry [12].

These strategies not only facilitate compliance with environmental regulations but also directly and comprehensively address the growing challenges faced by the industry. However, despite their significant advantages, such technologies may introduce new chemical and operational risks that must be rigorously assessed. To address this issue, risk analysis methodologies such as Event Tree Analysis (ETA) and Cause–Consequence Analysis (CCA) have been developed to identify the initial effects and their consequences, thus facilitating the implementation of mitigation measures [13,14].

Quantitative approaches such as the ALE (Annual Loss Expectation) model and the Courtney method have also been applied, allowing for risk prioritization and cost–benefit analysis based on numerical estimates such as frequency and probability. Additionally, specialized methods like FMEA/FMCEA, ISRAM, and CRAMM FMEA/FMCEA prioritize failures based on their critical impact and help select appropriate controls for mitigation. While all these methods are useful, they share common limitations such as a dependency on reliable data, the requirement for advanced tools, and subjective interpretation. They can also be labor-intensive, particularly in complex systems or during early design phases, making the selection of an appropriate method a challenging task [15].

In contrast, the Inherent Safety Index (ISI) methodology used in this study is not only a valuable tool for identifying and quantifying risks during the early design stages and in established processes, but it also enables a quantitative characterization of the inherent risks associated with the PVC production system integrated through mass and energy strategies under a zero-liquid-discharge (ZLD) approach. Although ISI has been applied to multiple chemical processes, there is limited documentation exploring its application in PVC plants that implement internal effluent treatment and circular production criteria. In this regard, the present study applies ISI to thoroughly characterize the critical areas of each stage in the suspension polymerization process of PVC, providing an accurate picture of the system’s internal safety and the specific features associated with zero discharge. This study aims to contribute valuable information for incorporating water sustainability criteria and optimizing the design of circular processes.

Developed in 1999 by Anna-Mari Heikkilä, the ISI uses pre-established scores and a “worst-case scenario” approach to determine the most critical value for each sub-index, based on the premise that inherently safe design eliminates hazards rather than mitigating them through additional controls. This index evaluates the process by dividing it into two main categories: the Inherent Chemical Safety Index, which analyzes material properties, and the Inherent Process Safety Index, which assesses inventory, temperature, pressure, and structural safety. This approach enables early hazard identification and the implementation of preventive measures in process design [16].

In addition to the above, the integration of computer-aided process engineering (CAPE) tools further strengthens the approach, enabling the modeling and optimization of various operating conditions. These tools facilitate the identification of risks and the selection of safer alternatives in the design of chemical processes by providing an analytical framework for managing multiple variables, thereby reducing reliance on extreme control measures to enhance safety [17].

Within the suspension process for PVC production, vinyl chloride monomer (VCM) represents a significant hazard due to its toxicity and emissions during stages such as drying and recovery [18]. For this reason, several technologies have proven useful in assessing specific aspects. The Layer of Protection Analysis (LOPA) is a semi-quantitative technique that identifies accident scenarios, quantifies their probability, and evaluates the effectiveness of independent protection layers (IPLs) applied to minimize risks, such as material leaks and uncontrolled reactions. It is based on well-established process risk analysis methods and uses an analytical approach to determine the adequacy of the protection layers employed for risk mitigation [19].

Markowski et al. [20], in a study, applied a modified version of this analysis called dLOPA to evaluate the risks in industrial drying processes, including the drying of PE and PVC in a fluidized bed dryer. In that study, they analyzed the likelihood of effective ignition sources and the severity of dust explosions, concluding that the implementation of IPLs is key to mitigating such risks. These results highlight the value of LOPA in risk assessment within PVC production and suggest its potential applicability in other critical areas of the process, such as wastewater recovery.

Similarly, the Hazard and Operability Analysis (HAZOP), which is widely used in complex chemical plants to control deviations in process variables that could lead to major incidents, was applied in a study on PVC production using sign-directed graphs (SDG-HAZOP). This study identified 36 critical failure propagation pathways, showing that valve-opening errors can lead to leaks of chlorine and other toxic compounds, thereby compromising process safety. Furthermore, the study concluded that this method partially automated risk analysis, improving accuracy in fault identification and reducing reliance on manual assessments [21].

However, despite the effectiveness of these methodologies, such studies do not comprehensively address safety within wastewater regeneration systems in PVC production, nor do they fully evaluate operating conditions, equipment, or emissions in the various critical sections of the process. This is where Inherent Safety Index (ISI) analysis plays a fundamental role, as it assesses process design from a broader perspective, identifying and minimizing risks throughout all stages. Therefore, this study conducts an inherent safety evaluation of the PVC production process with mass and energy integration, using the ISI framework and CAPE methodology to identify the main sources of risk. It analyzes both the impact of wastewater regeneration on process safety and the risks in critical stages such as reaction, VCM recovery, and resin purification, providing a comprehensive approach to make processes safer and more sustainable.

## 2. Materials and Methods

### 2.1. Process Description

**Figure 1:**
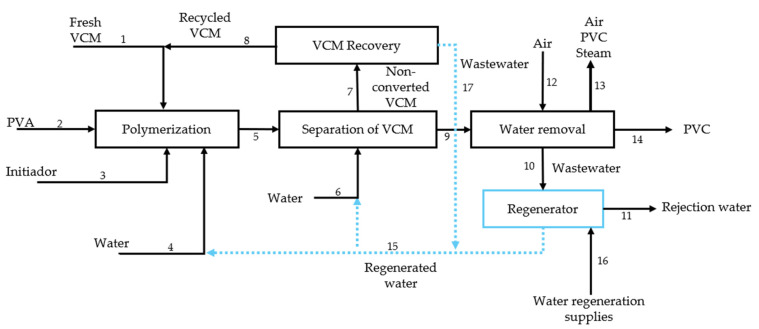
Flow diagram of the PVC suspension process with wastewater regeneration.

The PVC production process is optimized through various strategies aimed at reducing water and energy consumption, including wastewater reuse and material recovery. In this study, to define the baseline conditions and collect real data, the simulation model developed by Aguilar-Vásquez et al. [22] is used as a reference. This model is built using real operational data from industry, obtained under confidentiality agreements and supported by the specialized literature. Figure 1 illustrates this process.

Based on this model, the operating parameters are established, and the process operation sequence is described below. At the start of the operation, the reactor is fed with fresh and recycled VCM in a ratio of 80:20. VCM is polymerized in an aqueous solution using poly(vinyl alcohol) (PVA) as a stabilizer and a peroxide initiator (3-hydroxy-1,1-dimethylbutane-2-ethyl-2-methylheptane) at 70 °C and a pressure of 10 kgf/cm^2^. Since the reaction is exothermic, a cooling system is required to maintain stable conditions.

The conversion of VCM to PVC is approximately 85%, resulting in a suspension containing solid polymer particles, water, stabilizer, initiator, and unreacted monomer. To separate the PVC, the unreacted monomer is removed in two stages. In the first stage, the mixture is gasified in a flash tank where the pressure is reduced to 1.8 kgf/cm^2^, eliminating 95% of the residual VCM. In the second stage, a desorption tower with steam at 225 °C and 14 kgf/cm^2^ reduces the VCM concentration in the final PVC to less than 1 ppm, which is the internationally accepted limit [23,24].

After cooling, compression, and condensation, the VCM is recirculated to the reactor. The drying phase follows the separation of PVC. First, a centrifuge removes 75% of the water from the polymer paste. The remaining moisture is reduced in a dryer using hot air at 250 °C, which utilizes energy recovered from the centrifuge stream of the desorption tower to remove water, thereby reducing the fuel consumption of the burner [24].

Finally, the dry material passes through a cyclone and a bag filter, which capture fine polymer particles, ensuring a recovery efficiency of 99%. The wastewater generated during centrifugation and other process streams is directed to a regeneration system, where physicochemical treatment—including coagulation, flocculation, and clarification—is carried out using an aluminum-based coagulant that achieves a 99% removal efficiency. Subsequently, the stream is cooled to 35 °C before entering a biological system composed of anaerobic and aerobic reactors for PVA degradation [23]. The treated stream then passes through a reverse osmosis unit, modeled in Aspen Plus, where impurities are removed to enhance the quality of the reclaimed water, which is then recirculated to the reactor and boiler.

### 2.2. Inherent Safety Index

The Inherent Safety Index (ISI) is a tool used to assess the inherent safety level of a chemical process, emphasizing the elimination or reduction of hazards at their source [25]. Trevor Kletz and colleagues introduced this concept in the 1970s, following the Flixborough disaster [26]. This analysis consists of two main sub-indices that reflect different aspects of the process: the first is the Inherent Chemical Safety Index, which analyzes the chemical properties of the substances involved and their potential to cause hazardous incidents [16]. The second is the Inherent Process Safety Index, which evaluates the operational and design conditions that influence process safety [17]. The ISI is calculated using the following equation:

(1) $$I_{TI}=I_{CI}+I_{PI}$$

where the Total Inherent Safety Index ($I_{TI}$) is the sum of the Inherent Chemical Safety Index ($I_{CI}$) and the Inherent Process Safety Index ($I_{PI}$). If, at the end of the study, the process has a value greater than 24, it would indicate that it is inherently unsafe [27].

**Figure 2:**
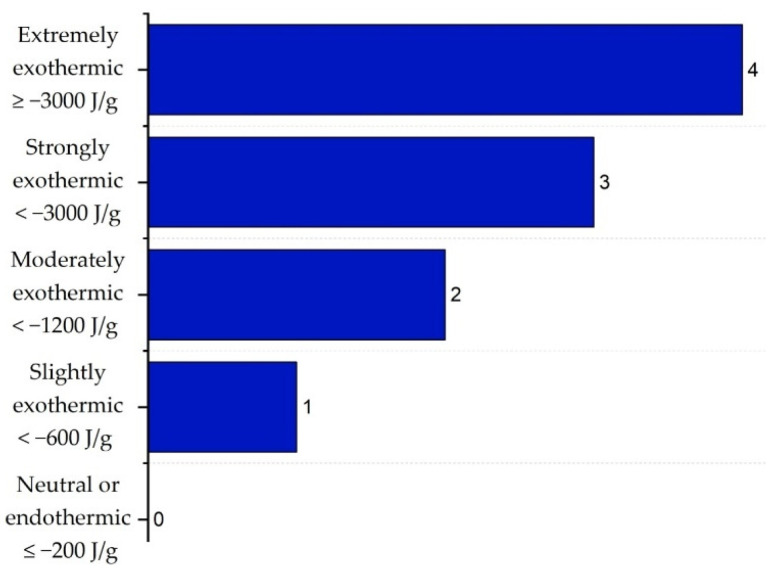
Determination of chemical reactivity sub-index.

**Figure 3:**
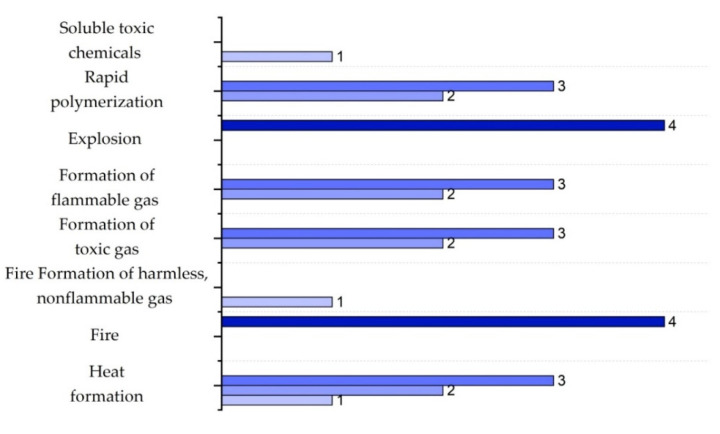
Determination of the chemical interaction sub-index.

**Figure 4:**
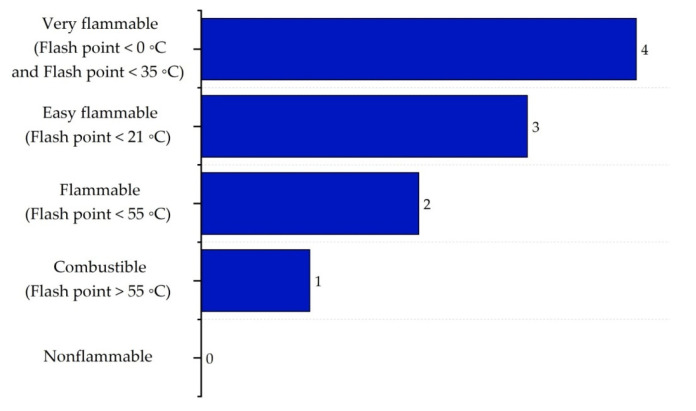
Determination of the flammability sub-index.

**Figure 5:**
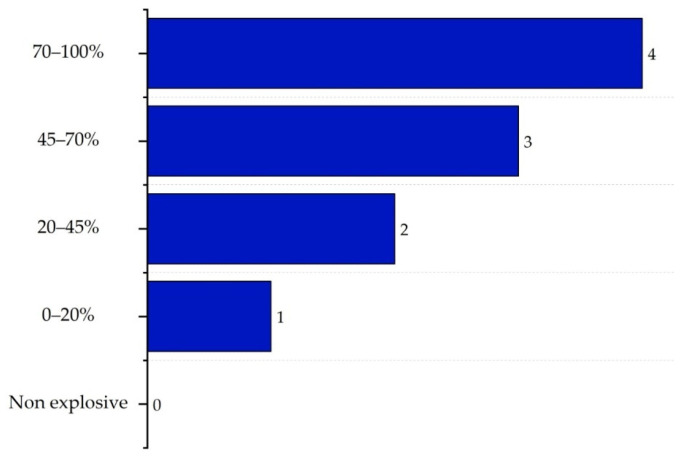
Determination of the explosivity sub-index.

**Figure 6:**
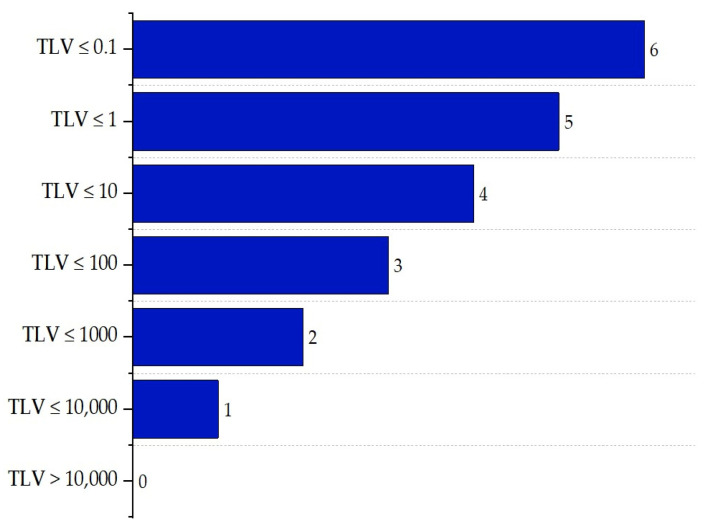
Determination of the toxic exposure sub-index.

**Figure 7:**
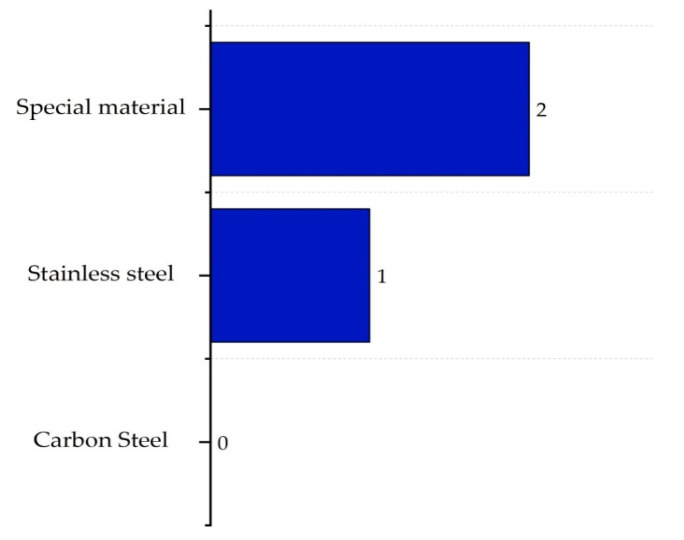
Determination of the corrosiveness sub-index.

**Figure 8:**
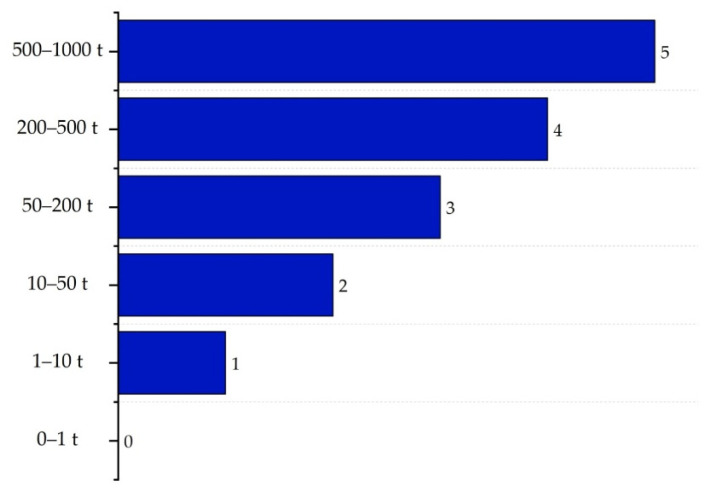
Inventory sub-index determination for ISBL.

**Figure 9:**
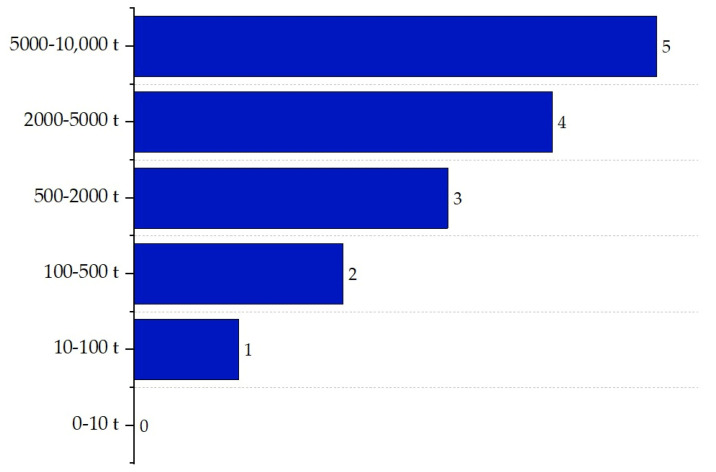
Inventory sub-index determination for OSBL.

**Figure 10:**
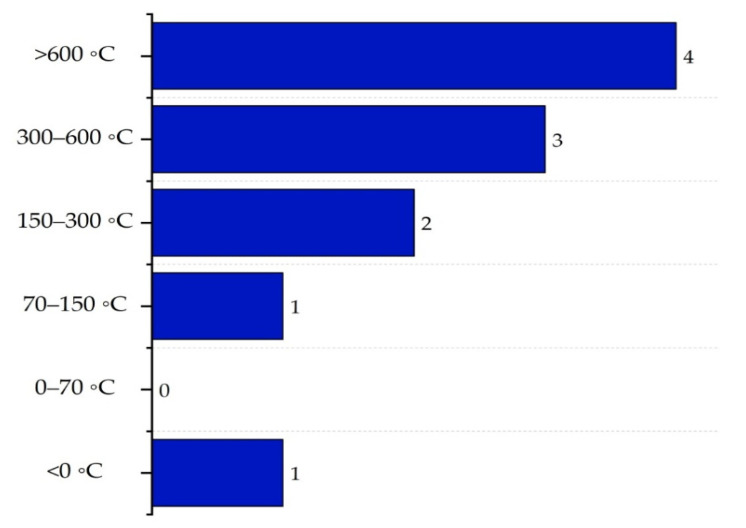
Determination of the process temperature sub-index.

**Figure 11:**
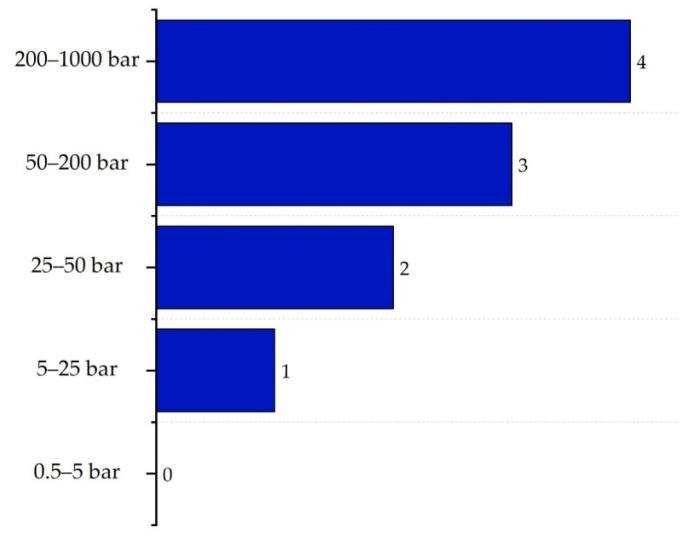
Determination of the process pressure sub-index.

**Figure 12:**
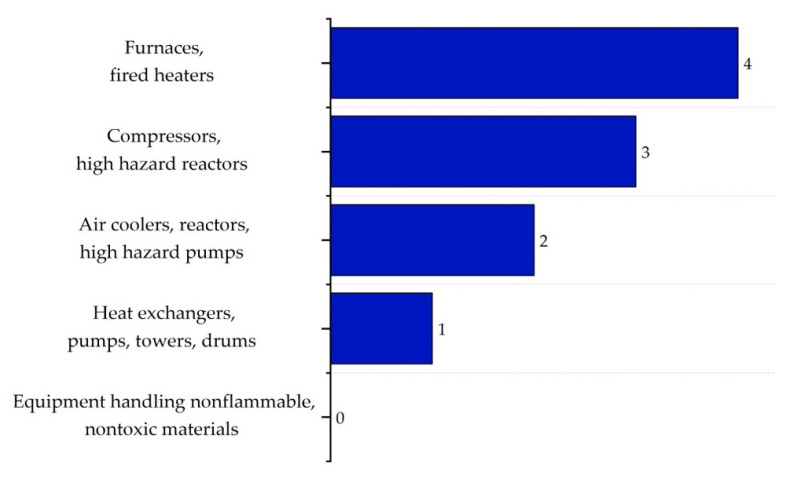
Equipment safety sub-index scores for ISBL.

**Figure 13:**
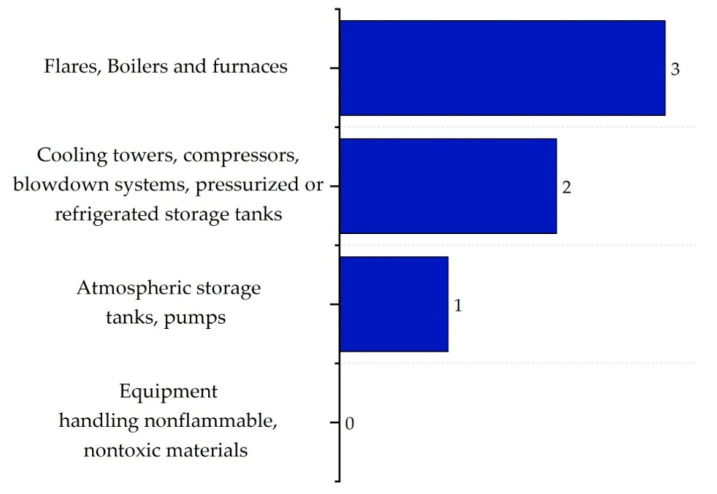
Equipment safety sub-index scores for OSBL.

**Figure 14:**
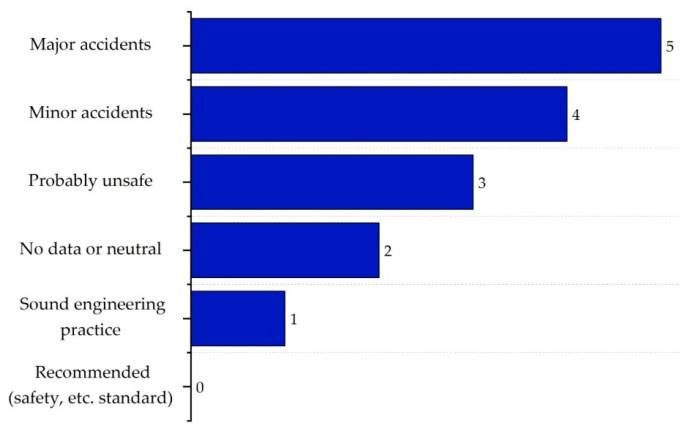
Safe process structure sub-index values.

It is important to highlight that the scoring ranges and evaluation criteria for each sub-index, represented in Figure 2, Figure 3, Figure 4, Figure 5, Figure 6 and Figure 7 (chemical safety sub-indices) and Figure 8, Figure 9, Figure 10, Figure 11, Figure 12, Figure 13 and Figure 14 (process safety sub-indices), are based on the methodology and tables published by Heikkilä et al. (1999) in their study “Inherent Safety in Process Plant Design: An Index-Based Approach” [16]. When assigning these scores to the process, operational conditions are considered, and the worst-case scenario is always assumed, as the ISI quantifies the inherent safety of the process based on the maximum potential risk posed by the plant.

### 2.3. Inherent Chemical Safety Index

According to the author, the Inherent Chemical Safety Index ($I_{CI}$) is represented by Equation (2) and measures the safety of a process based on the characteristics of the chemical compounds. It evaluates factors such as reactivity, flammability, explosiveness, toxicity, and corrosiveness [16].

(2) $$\;I_{CI}=I_{RM,max}+I_{RS,max}+I_{INT,max}+{\left(I_{FL}+I_{EX}+I_{TOX}\right)}_{max}+I_{COR,max}$$

Each sub-index is described as follows: $I_{RM,max}\;$ and $I_{RS,max}$ refer to the energy released or absorbed during the reaction, which is measured through the enthalpy of both primary and secondary reactions.

To determine the standard enthalpy of reaction ($\Delta H{{}^{\circ}}_{rxn}$), the equation based on Hess’s law is used. This involves subtracting the standard enthalpies of the formation of the reactants from those of the products, allowing for the calculation of the overall enthalpy change in the reaction using data from the literature.

$$\Delta H{{}^{\circ}}_{rxn}=\sum \Delta H{{}^{\circ}}_{f\;}\left(products\right)-\sum \Delta H{{}^{\circ}}_{f}\left(reactives\right)$$

According to Heikkilä et al. (1999) [16], the intensity of the heat released is assigned a score on a scale from 0 to 4, where lower values correspond to neutral or endothermic reactions and higher values to exothermic reactions. These values are presented in Figure 2.

The chemical interaction sub-index $I_{INT,max}$ refers to unintended reactions between process substances or with construction materials, which can generate excessive heat, fires, or undesired polymerization. The scores for this sub-index range from 1 to 4, depending on the risk level of the event: the most critical cases, such as fires and explosions, receive the highest score (4 points); moderate-risk events, such as the release of toxic or flammable gases and rapid polymerization, receive intermediate scores (2–3 points); and the least hazardous interactions, such as the generation of non-flammable and harmless gases, are assigned the lowest score (1 point). Figure 3 shows the distribution of these scores [16].

The flammability sub-index $I_{FL,max}$ is evaluated based on the flash point and explosive limits, with scores ranging from 0 for non-flammable substances to 4 for highly flammable substances, as shown in Figure 4 [16].

The explosivity sub-index $\left(I_{EX,\max}\right)$ expresses a substance’s tendency to form an explosive mixture with air and is calculated as the difference between the upper and lower explosive limits of the substance (UEL%–LEL%). Scores range from 0 for non-explosive substances to 4 for substances with a high risk due to their wide explosivity range (70–100%). The corresponding scores are shown in Figure 5 [16].

The toxicity sub-index $I_{TOX,max}$ is based on Threshold Limit Values (TLVs), which indicate the maximum permissible exposure levels of a substance over an 8 h period. Scores for this sub-index range from 0 to 6 points, depending on the hazard level of the substance: compounds with very high TLVs (TLV > 10,000) receive 0 points, while those with TLVs ≤ 0.1 receive the highest score (6 points), reflecting a higher toxic hazard. The scoring criteria are illustrated in Figure 6 [16].

Finally, the corrosivity sub-index $I_{COR,max}$ evaluates the potential of the compounds to degrade the integrity of the equipment. As more resistant materials are required, the index score increases. This evaluation is performed for all process streams, taking the worst case as the reference for determining the final value. The corresponding scores are shown in Figure 7 [16].

### 2.4. Inherent Process Safety Index

The purpose of the Inherent Process Safety Index is to assess how safe a process is from a design perspective, focusing on the critical aspects of operability and equipment configuration, and considering factors such as inventory, temperature, pressure, equipment safety, and overall process structure. The index is calculated using Equation (3), as described by Heikkilä et al. (1999) [16].

(3) $$\;I_{Ps}=I_{I}+I_{T,max}+I_{p,max}+I_{EQ,max}+I_{ST,max}$$

This indicator distinguishes between Inside Battery Limits (ISBL)—areas with smaller equipment, closer proximity, and a higher risk due to concentration—and Outside Battery Limits (OSBL)—areas with larger inventories but lower inter-device risk due to greater spacing [16].

Each sub-index is described as follows:

The inventory sub-index ($I_{I}$) estimates the total amount of material, considering mass flows and residence times, and poses a higher risk within ISBL. According to Heikkilä et al. (1999) [16], in the ISBL area, the score varies based on the amount of material accumulated in process equipment such as reactors or distillation columns—the larger the inventory, the greater the risk. Scores range from 0 for inventories of less than 1 ton to 5 for inventories between 500 and 1000 tons. The scoring criteria for this sub-index are presented in Figure 8.

Although OSBL (Outside Battery Limits) areas are not directly part of the core process, they also influence operational safety. In this context, the impact of larger material inventories on process safety is considered, particularly in equipment such as large-capacity storage tanks. The scores for this sub-index range from 0 for inventories of less than 10 tons to 5 for inventories between 5000 and 10,000 tons. Figure 9 illustrates the scores assigned to these inventory levels [16].

The temperature sub-index ($I_{T,max}$) analyzes the thermal risk of the system based on the maximum temperature that could compromise the integrity of construction materials (°C). According to Heikkilä et al. (1999), the score ranges from 0, where the risk is very low (between 0 and 70 °C), to 4 in critical conditions, i.e., when temperatures exceed 600 °C. Figure 10 presents the classification according to these criteria.

The pressure sub-index ($I_{p,max}$) considers the potential energy associated with leakage under normal operating conditions. Scores range from 0—indicating minimal risk (between 0.5 and 5 bar)—to 4, corresponding to high danger levels (between 200 and 1000 bar). The classification is presented in Figure 11 [16].

The equipment safety sub-index ($I_{EQ,max}$) assesses the risks associated with equipment within ISBL and OSBL areas, considering the risk of catastrophic failure, temperature extremes, and potential leaks. Equipment such as furnaces and heaters typically receive high scores due to their inherent risk, while equipment handling non-flammable and non-toxic materials present lower risks. In their study, Heikkilä et al. (1999) [16] provide a detailed explanation of this categorization, stating that equipment involved in high-risk operations tends to receive higher scores. The corresponding scores for ISBL equipment are shown in Figure 12.

For OSBL equipment, risks are assigned lower scores due to their lower impact compared to ISBL. OSBL equipment, often designed with larger clearances, tends to be safer. The scores for this sub-index are presented in Figure 13 [16].

Finally, the safe structure sub-index ($I_{ST,max}$) is based on recommendations and accident data, considering which designs are safer based on historical information and best practices [16]. The scores for this sub-index are shown in Figure 14.

The inherent safety analysis is carried out with the objective of identifying and reducing or eliminating, from the early stages such as design, the most significant risks of the process based on its basic characteristics, rather than relying on later control measures. This idea was introduced by Trevor Kletz in 1977 under the motto “what you don’t have, can’t leak” [28]. This approach is essential from the earliest design phases, such as the selection of raw materials and technologies or the configuration of reaction stages, since this is when the most critical process decisions are made and they have a direct impact on the risk levels that operations may present in the future [26].

In contrast to traditional methodologies, according to Abedi et al. [28], the Inherent Safety Index “fundamentally differs from secondary prevention and accident mitigation”. In other words, this index seeks to avoid the need for adding protective layers by addressing the problem at its root, making it a proactive and effective tool for primary prevention.

In this study, the index is used to identify the risks and critical stages within the PVC suspension production process. The results allow us to determine whether the process is inherently unsafe, as Heikkilä mentions, if the score exceeds 24, or safe, if it falls below this value [27]. These findings not only help evaluate the process’s safety, but also support recommendations for improvements that align technical and economic considerations.

## 3. Results

The Inherent Safety Index (ISI) methodology has been applied to various chemical processes; however, in the case of the suspension PVC production process with mass and energy integration and wastewater regeneration under a zero-discharge approach, it has been explored very little. This study aimed to provide a detailed analysis of the inherent safety in this type of system.

**Figure 15:**
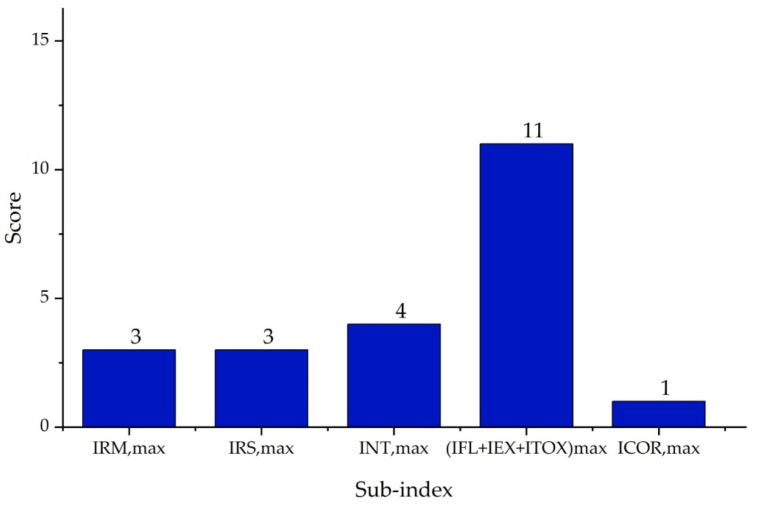
Chemical sub-indices of the PVC suspension process.

**Figure 16:**
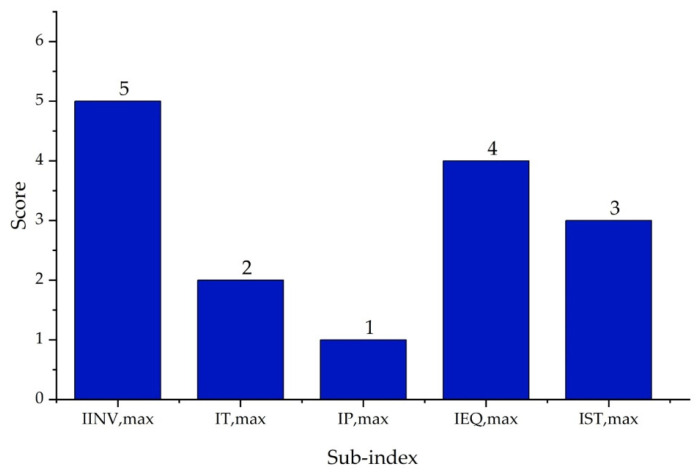
Process safety sub-indices for PVC suspension process.

**Figure 17:**
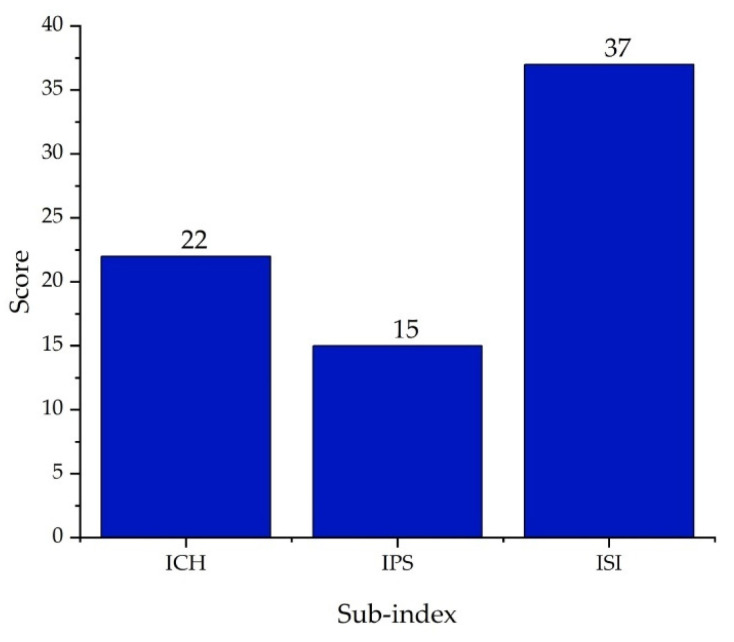
Total Inherent Safety Index for the PVC suspension process.

The scores of the sub-indices (Figure 15, Figure 16 and Figure 17) were assigned considering the worst-case scenario, as this reflects the highest risk the plant may present and serves as the basis for quantifying its inherent safety.

Figure 15 below presents the values obtained for the Inherent Chemical Safety Index, calculated for the PVC production process with mass and energy integration and wastewater regeneration. The main reaction was the polymerization of vinyl chloride monomer (VCM), which was highly exothermic with a $\Delta H_{R}=-1600\;\mathrm{kJ}/\mathrm{kg}$ and therefore required precise temperature control through constant cooling to avoid accidents. The score assigned to this sub-index $(I_{RM,max}$) was 3 points.

Regarding the secondary reactions identified in the regeneration system, two were found to be related to the biodegradation process of poly(vinyl alcohol) (PVA). To assign their respective scores $I_{RS,max}$, the enthalpy of the formation of PVA was first calculated based on its monomer unit (vinyl alcohol), assuming a polymerization degree of 1000 and summing the polymerization heat released per repeating unit. Once this value was obtained, the standard reaction enthalpy ($\Delta H{{}^{\circ}}_{rxn}$), was determined using Hess’s law.

The first reaction corresponded to the anoxic degradation of PVA, for which a reaction enthalpy of −156.5 kJ/kg was obtained. As it was a thermally neutral reaction, its score was 0. The second reaction corresponded to the aerobic oxidation of PVA, with a reaction enthalpy of −2304 kJ/kg, classifying it as extremely exothermic and assigning it a score of 3 points in the $I_{RS,max}$ sub-index.

For the toxicity, explosivity, and flammability ratings for each substance, information was obtained from the online Safety Data Sheets (SDSs) on the Chemical Safety website, which provide essential safety data (EMS) [29], as well as from the NIOSH Pocket Guide to Chemical Hazards (NPG), which contains general industrial hygiene information for hundreds of chemicals [30].

In terms of hazard, although several substances were involved in the process, VCM stood out as the compound with the greatest impact due to its combination of hazards. Firstly, it has a wide explosive hazard range (UEL%–LEL%) of 29.40%. It is highly flammable, which is why it received the highest rating in this category: 4 points. It can partially decompose in the presence of sodium or potassium hydroxide, and when in contact with air, it forms explosive polymeric peroxides. Additionally, it can polymerize rapidly due to heat or the presence of strong oxidizing agents.

On top of this is its toxicity. During combustion, it generates highly toxic products such as hydrogen chloride, phosgene, carbon dioxide, and carbon monoxide, which makes it necessary to strictly control the oxygen content during storage and handling [31]. With a Threshold Limit Value (TLV) of ≤1 ppm, it obtained the maximum toxicity score, which is 5.

Other substances also posed safety concerns, such as Sodium hydroxide (NaOH) and Sodium hypochlorite (NaOCl), which, due to their toxic impact, received 4 points in this category, as well as PVC, which scored 5 points, just like VCM. Sodium hypochlorite is unstable and decomposes upon contact with acids, sunlight, and certain metals, releasing corrosive gases such as chlorine and oxygen, which increase the risk of combustion in the presence of flammable materials. Under certain conditions, it can form highly hazardous compounds, such as nitrogen trichloride (NCl_3_), which is explosive [32,33].

Sodium hydroxide, on the other hand, is an extremely corrosive material. It reacts exothermically with acids, releasing heat that can lead to boiling and dangerous splashing. Although it is not flammable, when it comes into contact with metals such as aluminum and zinc, it releases hydrogen gas, which is highly flammable. Furthermore, its interaction with certain chemicals can trigger violent reactions, increasing the risk of accidents during processing [32,34].

Finally, PVC, although more stable than the other compounds, is not exempt from posing a risk, as it can undergo thermal decomposition at high temperatures, releasing hazardous and corrosive gases such as hydrogen chloride, and forming polyenes, which present additional fire hazards [35]. Taking these considerations into account, Table 1 presents a comparison of the main safety risks associated with the primary substances in the process.

To better visualize the assignment of scores to substances according to their chemical safety parameters, Table 2 is presented.

The combination of these substances with the operating conditions can generate synergistic effects that significantly increase the overall risk of the process. The high exothermicity of the main reaction, for example, combined with degradation induced by oxidizing agents such as sodium hypochlorite (NaOCl) and sodium hydroxide (NaOH), accelerates the deterioration of metallic system components such as heat exchangers, valves, and sealing joints—contributing simultaneously to the risk of overpressure and the mechanical failure of equipment [36]. Furthermore, as previously mentioned, the presence of NaOH can lead to the release of hydrogen gas, creating an inherently flammable atmosphere even in the absence of ignition sources [37].

In such conditions, a vinyl chloride monomer (VCM) leak would exponentially increase the potential for a catastrophic scenario, due to its high toxicity (TLV ≤ 1 ppm) and wide explosive range (29.4%). This was demonstrated in the incident documented by Ogle et al. (2004) [38], where the release of overheated VCM in a poorly designed tank led to instantaneous vaporization and an overpressure explosion—caused by several factors, including the synergistic interaction between exothermicity, chemical composition, and elevated pressures. Similarly, in the ARIA (2014) case, it was shown that a minor mechanical failure was drastically amplified when internal pressure, accumulation of explosive gases, and highly reactive substances such as VCM, NaOH, and NaOCl were involved [39].

Given the synergistic behavior between temperature, pressure, and chemical agents, material selection becomes a critical aspect of inherent safety, as the use of inadequate materials has previously been responsible for serious failures in PVC plants, such as leaks and explosions [38,39].

For this PVC production process, stainless steel was selected. Its composition—including iron, chromium, and other elements such as nickel and molybdenum—forms an adherent chromium oxide layer that protects the material in oxidizing environments and allows it to withstand high temperatures and pressures while maintaining its mechanical strength and rigidity. Additionally, it prevents unwanted reactions that could affect the polymer quality, avoids accumulation on surfaces, and facilitates the cleaning process [40,41,42]. This selection becomes even more relevant considering, for instance, that the corrosion rate increases exponentially with temperature, approximately doubling with every 10 °C rise [43]. Moreover, high pressures enable corrosive species to penetrate more easily into microcracks under HPHT (high-pressure, high-temperature) conditions, which accelerates embrittlement and crack propagation in equipment [44]. When these conditions are combined with constant mechanical stresses in a corrosive environment—such as the one present in this process—they result in stress corrosion cracking (SCC), a failure mechanism that requires the simultaneous presence of mechanical stress, chemical attack, and material susceptibility [45]. Finally, corrosion fatigue, which results from repeated loading in an aggressive medium, causes much more severe damage than when each factor acts independently, ultimately reducing the service life of the process components [46].

For all these reasons, the equipment corrosivity sub-index $(I_{COR,max}$) received a score of 1, reflecting the high effectiveness of stainless steel in mitigating these synergistic degradation mechanisms.

The maximum temperature sub-index $(I_{T,max}$) was rated at 2, as the drying zone heater reached 250 °C. Regarding the pressure sub-index $(I_{P,max}$), the highest recorded pressure was 13.73 bar in the boiler, which corresponded to a score of 1.

In this case, although the conditions were not extreme when considered individually, their combination with volatile substances such as VCM created a risk window [38]. High pressures not only contribute to undesirable reactions but also accelerate the formation of microcracks in materials, even under moderate conditions.

Regarding the wastewater regeneration system, although the operating temperature was moderate (35 °C), it was still necessary to evaluate the synergistic chemical–mechanical effects that influenced material durability and, consequently, process safety.

This stage involved physical–chemical and biological treatments that included VCM, initiators, aluminum-based coagulants, and PVA. According to the Cole-Parmer chemical compatibility reference, these substances only have minor effects on the material, causing slight corrosion or minimal discoloration of the alloy [47]. However, these effects can promote the formation of microcracks, especially when continuous flow stresses are also present [45].

Therefore, the protective chromium oxide layer naturally formed on stainless steel plays a key role in preserving the integrity of the equipment. Moreover, studies have shown that poly(vinyl alcohol) (PVA) is not only compatible with metals, but also exhibits potential as an anticorrosive agent, acting as a corrosion inhibitor and enhancing the stability of metallic coatings [48]. Thus, if PVA were used as a surface treatment inhibitor, it could reinforce passivation and help minimize residual attacks.

As a result, the compatibility of stainless steel in this part of the process justified assigning the corrosivity sub-index $(I_{COR,max}$) a score of 1.

The total process inventory, based on one hour of operation, reached an average of 3241.75 t/h, indicating a high accumulation of substances in the production areas. Although this volume is high, it is standard in the PVC industry; in fact, in the VCM production process, the inventory range is approximately 300 t/h [49]. Nevertheless, the presence of these large inventories under elevated operating conditions of temperature, pressure, and the use of flammable and corrosive agents constitutes a critical factor in the evaluation.

In this context, this explains why the area within the battery limits (ISBL), which included the main process units, reached the highest score of 5 in the ISBL inventory sub-index. This positioned it as one of the components with the highest risk in the process, second only to the toxicity sub-index. This score did not merely reflect volume; rather, it suggested a potential risk that arose when large quantities of PVC coexisted with moderate conditions of 250 °C and 13.7 bar.

On the other hand, in the areas outside the battery limits (OSBL), the same synergy between the large volumes of raw materials stored, the pressure and temperature variations, and the volatile chemical nature of most of the substances involved in this process—including initiators—justified an OSBL inventory sub-index score of 4. Figure 16 shows the corresponding values for the process safety of PVC suspension.

The equipment safety sub-index ($I_{EQ,max}$) reached the maximum score of 4 in the ISBL zones due to the inclusion of critical equipment such as the gasifier, reactors, and compressors. Although they operated under moderate conditions, they were simultaneously exposed to corrosion and stress corrosion cracking (SCC). In contrast, in the OSBL areas, equipment such as boilers and furnaces scored a 3, indicating a lower but still significant level of risk. However, it is worth noting that although these thermal systems were classified as risky, their use of non-flammable and stable energy carriers—such as water and air—made them safer.

For its part, the process structure safety sub-index ($I_{ST,max})$ was evaluated not only based on the operating conditions of the process but also by considering historical accident records in the industry. These records show that a lack of proper process control often leads to accidents or significant consequences, making this assessment essential in our case.

Although the operating conditions were moderate—such as in the reactor, which operated at 70°C and 10 kgf/cm^2^, and the desorption tower, which operated at 14 kgf/cm^2^ and 225 °C—they required strict control to ensure the stability and safety of the process and to meet operational requirements, such as achieving a monomer recovery efficiency of 95% (see Section 2.1).

Adding to this level of complexity is the wastewater regeneration stage, which includes physicochemical treatment (coagulation, flocculation, and clarification), biological treatment (anaerobic and aerobic reactors for the degradation of PVA), and a reverse osmosis stage. Interaction with these auxiliary systems can alter material flows and influence heat exchange operations. However, it also brings the advantage of effectively removing PVA and other impurities, reducing fouling problems in equipment and contributing to a more stable operation [50].

It is essential to assess their impact on the process configuration, as it is well known that in any industrial process, the integration of additional units presents challenges that—if poorly managed—could have significant consequences for process safety, whether due to design flaws or control failures.

Throughout industrial history, failures in equipment control and maintenance have been recorded as causes of serious consequences. Below, we describe some of these incidents that resulted in major catastrophes.

In 1977, a critical event occurred at the PRIMEX plastics factory in Puebla, Mexico. A vinyl chloride gas leak triggered a fire, followed by four explosions that destroyed the plant and caused numerous casualties. The scale of the disaster forced the evacuation of entire neighborhoods, while burning debris and explosion remnants affected surrounding areas—clearly demonstrating the risk of handling large volumes of flammable substances without proper controls [51]. After this accident, systems were implemented such as early gas leak detection, the retrofitting of storage tanks with double containment, and strict preventive maintenance protocols for critical equipment such as piping and valves.

Years later, one of the most severe incidents in the PVC industry occurred in 2004 at the Formosa Plastics plant in Illiopolis, Illinois. An explosion destroyed a PVC-1 production unit after the release of vinyl chloride—a compound which, as previously noted, is highly flammable. This accident resulted in the death of four workers, multiple injuries, and significant structural damage. Moreover, the plant’s water deluge system, designed to contain such material leaks, failed to operate properly, exposing serious deficiencies in its safety measures [52].

Another incident occurred on 20 August 2000, when a chlorine gas leak at the Solvay Indupa plant in Bahía Blanca posed a serious risk to the surrounding community. Despite the gas’s toxicity, no warning was issued to residents, revealing a critical disregard for safety protocols. The leak, which caused pressures between 4600 and 5200 kg/cm^2^, was due to the rupture of a 15-year-old pipe that had not been inspected or properly maintained for over a year. According to experts, a major tragedy was only avoided thanks to external factors—specifically, favorable wind conditions that diverted the toxic cloud toward a less populated area, mitigating what could have been a much larger catastrophe [53].

Although pressure and temperature in this process do not, by themselves, pose severe risks, the combination of flammable materials and any deficiencies in safety protocols could significantly multiply the hazards. For this reason, we assigned a score of 3 to the ($I_{ST,max}$) sub-index. While this score reflects a potentially unsafe process structure, it may be an overestimation. For instance, Carvalho et al. [49] actually rated the VCM production process as neutral, and Lozano assigned a score of 0 to the acetone production process [54]. Nevertheless, these accident reports highlight that the main concerns do not necessarily lie in the technical configuration of the process, but rather in the lack of control, maintenance, and effective protocols—which have been the root cause of chaotic events.

Finally, the overall assessment resulted in a total Process Safety Index ($\;I_{PI})$ score of 22, as shown in Figure 17, with the equipment and inventory sub-indices representing the highest risks within the process.

## 4. Discussion

Figure 17 shows that the PVC production process—integrated in mass and energy with water regeneration—has a Total Inherent Safety Index of 37. This value exceeds the safety threshold of 24 defined by the ISI methodology [16]. According to the criteria established by Heikkilä et al. (1999), this result indicates that the process is inherently unsafe, implying significant operational risks and requiring the implementation of mitigation measures, as will be discussed later [14].

Specifically, the Chemical Safety Index obtained a total score of 22, with VCM identified as the most hazardous substance. VCM is a highly flammable, explosive, and toxic gas. A leak of this material could cause fires, explosions, and severe toxic accidents, thereby increasing the risks associated with storage and handling in the industry [55]. Although other substances used in the process, such as NaOH and NaOCl, are not as critical as VCM, their toxicity and instability still contribute to the overall operational risk.

Since VCM was the main raw material in this process, its hazardous nature was a major concern. Therefore, the most effective way to reduce the risks associated with its use is to implement a rigorous safety system, along with careful planning and proper management throughout all stages of the process. A similar observation can be made in the production of acetic acid through methanol carbonylation, which reached a total of 10 points in the combined sub-indices ${\left(I_{FL}+I_{EX}+I_{TOX}\right)}_{max}$.

Another reason for the high index in our case study was the presence of secondary reactions within the regeneration system. Specifically, the biodegradation of poly(vinyl alcohol) (PVA) gave rise to two reactions: one was anoxic and thermally neutral, while the other was aerobic and considered extremely exothermic. This resulted in a greater potential for heat generation within the system, which could increase the risks of thermal instability and the propagation of incidents.

From a process safety perspective, an index score of 15 was obtained. The least significant impacts were observed in the pressure and temperature conditions, which were moderate. The temperature reached 250 °C, while the pressure was 13.73 bar. These conditions contributed to a favorable score in terms of operational safety. The safe structure index, for its part, revealed that the process presented a moderate risk level. This was mainly due to the improper handling of substances such as VCM, which, as previously discussed, could lead to a catastrophe. However, it is important to note that there were phases of the process where this risk was mitigated.

Regarding the critical points identified within the facility, the most significant issues were found in equipment and inventory. The inventory reached 3241.75 t/h, a substantial volume that highlighted the scale of the plant’s production. This is a distinctive size within the PVC industry, with a total production volume of 6963.5 t/h [56,57]. Therefore, although the value is high, it is standard for PVC and VCM production. Naturally, such a considerable quantity demands stricter management.

In the equipment sub-index, the most hazardous components included the reactors and compressors located within the ISBL areas, while in the OSBL areas, the main safety concerns involved boilers and heaters—devices that operate under extreme thermal conditions and require continuous monitoring. However, the latter systems use heat transfer methods involving water and air, which reduce their overall hazard level despite the scores assigned to them [58].

Regarding recommendations to reduce the risks in this process, Gonzalez-Delgado et al. [58] propose optimizing the storage and distribution of VCM, reducing its presence in high-exposure areas, improving the isolation of zones with high VCM concentrations, and relocating critical equipment to lower risk areas when feasible. These recommendations align with the implementation of a comprehensive safety system that not only addresses the risks associated with handling hazardous substances such as VCM but also ensures the protection of personnel and infrastructure. Additionally, they suggest modifying operating conditions—such as pressure and temperature—to minimize inherent process risks. To ensure the process remains feasible, they recommend conducting a cost–benefit analysis, allowing for gradual and optimal implementation, particularly during the final stages of process design.

Further recommendations include incorporating real-time sensors and alarm systems, enhancing staff training in risk identification and emergency response, ensuring safety protocols are regularly updated, maintaining proper ventilation in confined areas with high VCM concentrations, and optimizing preventive maintenance practices to ensure the integrity of pipelines and connections.

The incorporation of real-time monitoring and alert systems has proven to be particularly effective in preventing incidents and enhancing overall process safety.

An example of this is the company Vulcano Plástico, which implemented these monitoring and control systems in its extrusion line. This resulted in reduced production times and improved thermal control of the machinery, thereby optimizing the operation of the extrusion line and enabling the real-time visualization of process conditions [59].

On the other hand, a key—if not mandatory—aspect is the training of personnel in risk detection and emergency response. This not only helps reduce the likelihood of accidents but also fosters a strong safety culture within the plant. This need is underscored in an analysis of a PVC extrusion plant, which highlights the importance of training under standards such as OHSAS 18001 to minimize workplace incidents [60].

Similarly, it is essential to ensure proper ventilation in areas with high concentrations of vinyl chloride monomer (VCM), as organizations such as the ATSDR have set exposure limits to reduce the risks of accumulation in confined spaces. Therefore, adequate airflow in such areas is critical [61].

Preventive maintenance optimization is also vital to avoid accidents that may compromise workers’ health. It ensures that pipelines and connections remain in proper condition, as recommended in a study on working conditions in plastics plants [62].

Finally, keeping safety practices up to date is fundamental for the continuous improvement of occupational risk management and the effectiveness of preventive measures. In fact, ISO 45001 establishes that it is the organization’s responsibility to carry out this task periodically to identify improvement opportunities, implement corrective actions, and prevent the recurrence of accidents [63].

## 5. Conclusions

The inherent safety analysis of the suspension PVC production process showed a Total Inherent Safety Index of 37, exceeding the acceptable limit defined by the ISI methodology [16], indicating that the system is inherently unsafe. Despite the environmental benefits derived from water use optimization, significant risks persist in terms of inherent safety.

The Chemical Safety Index reached a value of 22, highlighting vinyl chloride monomer (VCM) as the most hazardous substance due to its toxicity, flammability, explosiveness, and dangerous chemical interactions with air and oxygen—compounds that are unavoidable in the process.

Beyond its intrinsic hazard, these interactions lead to critical secondary consequences: the thermal and chemical degradation of both vinyl chloride and its polymer (PVC) due to the action of free oxygen, which affects polymerization kinetics and particle size distribution by acting as both a radical initiator and inhibitor [64]; the thermal dehydrochlorination of PVC, which releases hydrogen chloride (HCl) and forms conjugated polyenes, compromising the material’s integrity and physical properties [65]; and the volatilization of VCM in wastewater, with its presence detected in treatment systems, including anaerobic digesters, and released into the environment via valves and pipe joints, evidencing its persistent presence and migration into the surroundings [66].

Additionally, the Process Safety Index, with a value of 15, revealed critical risks related to inventory management and the operation of equipment such as reactors, boilers, and compressors.

Although the integration of water regeneration improves the process’s sustainability, it also introduces new operational challenges, such as the formation of secondary reactions with high thermal potential, increasing system complexity and thermal instability. Therefore, it is essential to strengthen process safety measures: optimize the storage and handling of VCM with proper containment; enhance the monitoring and maintenance of key equipment through real-time sensors; and regularly review and update safety protocols based on cost–benefit analyses and failure simulations when possible. This will enable a balance between environmental goals and operational stability, ensuring a viable, safe, and sustainable long-term process.

## Figures and Tables

**Table 1:** Chemical safety parameters of chemical substances [16].

Substance	Explosivity (UEL%–LEL%)	Flammability	Toxicity TVL (ppm)	Chemical Interaction
VCM	29.40%	Highly flammable	TLV ≤ 1	Explosion
NaOH	0	Non-flammable	TLV ≤ 10	Fire
NaOCL	0	Non-flammable	TLV ≤ 10	Explosion
PVC	0	Combustible	TLV ≤ 1	Explosion

LEL: lower explosive limit, UEL: upper explosive limit.

**Table 2:** Summary of scores by category for the main substances [16].

Substance	Explosivity (UEL%–LEL%)	Flammability	Toxicity TVL (ppm)	Chemical Interaction
VCM	2	4	5	4
NaOH	0	0	4	4
NaOCL	0	0	4	4
PVC	0	1	5	4

LEL: lower explosive limit, UEL: upper explosive limit.

## Data Availability

The data will be available upon reasonable request to the correspondence author (Á.D.G.-D.).

## Abbreviations

ETA	Event Tree Analysis
CCA	Cause–Consequence Analysis
CAPE	Computer-aided process engineering
ISRAM	Information Security Risk Analysis Method
FMEA	Failure mode and effects analysis
HAZOP	Hazard and Operability study
HRA	Health risk assessment
